# From innovation to implementation: a case report on virtual reality for chronic pain and psychological well-being in clinical practice

**DOI:** 10.3389/fpsyt.2026.1864629

**Published:** 2026-08-12

**Authors:** Naiomi Rivera-Rivera, Janet Lopez, Nicole Haberland, Brendaliz Diaz-Guevara

**Affiliations:** 1 Behavioral Health & Mental Health Service, VA Caribbean Healthcare System, San Juan, Puerto Rico; 2 College of Nursing, University of Central Florida, Orlando, FL, United States; 3 College of Medicine, University of Central Florida, Orlando, FL, United States

**Keywords:** Caribbean, digital therapeutics, interdisciplinary pain rehabilitation program, mental health outcomes, non-pharmacologic intervention, veterans

## Abstract

**Background/objectives:**

Virtual reality (VR) is an emerging non-pharmacologic intervention for chronic pain management among Veterans. Although randomized controlled trials have demonstrated the efficacy of skills-based VR interventions such as RelieVRx for reducing pain and anxiety, less is known about how these interventions can be implemented within real-world interdisciplinary care settings in the Caribbean or how they influence patient engagement and psychological outcomes in routine practice. This case series aims to describe real-world implementation and patient-level outcomes with Veterans in the Caribbean.

**Materials and methods:**

This case report describes the adoption of RelieVRx into the Interdisciplinary Pain Rehabilitation Program (IPRP) at the VA Caribbean Healthcare System. Data from three bilingual Veterans with chronic pain who participated in the IPRP and completed 56 sequential RelieVRx sessions were analyzed for changes in subjective pain levels, anxiety, and depressive symptoms. Veterans’ qualitative experiences with the device were also assessed.

**Results:**

Clinical adoption of RelieVRx involved identification of appropriate clinicians, alignment with existing workflows, staff training and competency verification, a supervised trial period, standardized documentation of use, and ongoing performance monitoring. All three Veterans reported improvements in mood following RelieVRx use. Two Veterans reported reductions in subjective pain levels, while pain levels remained unchanged for one Veteran. Improvements in depressive and anxiety symptoms were observed in two Veterans, while qualitative reports suggested enhanced emotional regulation across all cases.

**Conclusions/discussion:**

RelieVRx appears to be a feasible adjunct to interdisciplinary pain care intervention. This report highlights real-world implementation and patient-level experiences rather than establishing causal effectiveness. In addition to perceived improvements in pain, Veterans reported benefits in mood. Participants also described sustained usefulness of the device beyond the structured 56-session intervention, extending into their daily lives.

## 1 Introduction

Chronic pain affects many Veterans in the United States and remains a challenging condition to manage using conventional approaches alone (1, 2). Non−pharmacologic therapies are increasingly recognized as essential components of comprehensive pain management, particularly amid ongoing efforts to reduce reliance on opioid analgesics (3). Virtual reality (VR) has emerged as a promising modality within this landscape, offering immersive, multisensory environments that can redirect attention, modulate pain perception, and support the acquisition of psychological coping skills (4, 5). Through vivid visual, auditory, and sometimes tactile simulations, VR can shift cognitive focus away from nociceptive input, resulting in measurable reductions in pain intensity and distress.

A growing body of evidence supports VR’s effectiveness across acute and chronic pain conditions (5–8). Skills−based VR programs that integrate cognitive behavioral therapy (CBT), mindfulness, and pain−education components have demonstrated clinically meaningful improvements in pain intensity, pain interference, and functional outcomes in randomized controlled trials (9). These interventions may be especially beneficial for individuals with mobility limitations, heightened anxiety, or those seeking non−pharmacologic alternatives (5). Although randomized controlled trials have demonstrated that skills-based VR interventions can improve pain and anxiety outcomes, less is known about how these interventions can be integrated into real-world interdisciplinary pain rehabilitation programs or how patients engage with them in routine clinical care. Understanding implementation processes and patient experiences is critical, particularly among Veterans with complex comorbidities (5).

Within military and Veteran populations, VR has been applied to improve function (10, 11), reduce procedural discomfort (12), and support treatment for post−traumatic stress disorder (13, 14). Early evidence also suggests benefits for pain and anxiety management (15). However, the extent to which VR interventions simultaneously improve pain and mental health outcomes among Veterans remains insufficiently examined. This gap is particularly important given the high prevalence of chronic pain and comorbid mental health conditions in this population, as well as the need for scalable, engaging, non−pharmacologic interventions that align with whole−person care frameworks.

In this case report, we sought to address this gap by documenting the implementation of RelieVRx for the first time within the VA Caribbean Healthcare System as part of the Interdisciplinary Pain Rehabilitation Program (IPRP). This initiative aimed to enhance psychological well-being and improve pain management among Veterans. The Caribbean context is particularly important, as Veterans in this region often experience unique healthcare challenges, including geographic barriers, limited access to specialized non-pharmacologic interventions, and a high burden of chronic pain and mental health comorbidities (16–18). Implementing scalable, technology-based interventions such as RelieVRx may help expand access to evidence-based care and support more equitable health outcomes in underserved settings. This case series contributes to the literature by illustrating real-world clinical implementation and patient engagement within an interdisciplinary pain rehabilitation program in the Caribbean.

## 2 Methods

The adoption of RelieVRx into the Interdisciplinary Pain Rehabilitation Program (IPRP) at the VA Caribbean Healthcare System followed a structured implementation process. Key components included identification of clinical gatekeepers, assessment of compatibility with existing workflows, clinician training and competency verification, a supervised trial period, standardized documentation of device use, and ongoing performance monitoring. In parallel, three case studies of Veterans enrolled in the IPRP were analyzed to evaluate changes in subjective pain levels, anxiety, and depressive symptoms following completion of the RelieVRx intervention. Inclusion criteria for this case series included (1): enrollment in the IPRP (2), diagnosis of chronic non-malignant pain (3), completion of the 56-session RelieVRx program, and (4) availability of pre- and post-intervention outcome data when feasible. In one case, standardized anxiety data (GAD-7) was not consistently collected as part of routine care; however, the case was retained to illustrate real-world implementation and patient experience. Excluding such cases would limit the ability to reflect actual implementation conditions. Adverse effects related to VR use, such as cybersickness, were monitored as part of this implementation.

Outcome measures were collected at baseline and post-intervention in accordance with routine clinical workflows; intermediate assessments were not conducted. Subjective pain levels were measured with the Pain, Enjoyment, and General Activity (PEG) questionnaire, a brief three-item scale that assesses pain intensity and its interference with daily activities and enjoyment of life; it has demonstrated good reliability, validity, and responsiveness in large samples of chronic pain patients (19, 20). The PEG is scored by averaging the items, resulting in scores ranging from 0 to 10.

Anxiety symptoms were measured with the Generalized Anxiety Disorder scale (GAD-7), a brief seven-item measure that assesses the presence and severity of anxiety symptoms and has demonstrated strong reliability and validity (21). The GAD-7 is scored by summing the items, with scores ranging from 0 to 21.

Depressive symptoms were measured with the Patient Health Questionnaire (PHQ-9), a brief nine-item scale assessing the presence and severity of depressive symptoms; it has also demonstrated strong reliability and validity (22). The PHQ-9 is scored by summing the items, with scores ranging from 0 to 27.

### 2.1 Settings and subject

The Interdisciplinary Pain Rehabilitation Program (IPRP) at the VA Caribbean Healthcare System is an intensive four-month program for Veterans with chronic pain. Veterans admitted to the clinic undergo physical therapy, recreational therapy, medication optimization, physiatry evaluation, social interventions, and psychological care. Patients were referred to the IPRP and, after an initial evaluation by the multidisciplinary team, were admitted and became eligible to use the device as part of the available psychological interventions.

RelieVRx is an FDA-authorized, self-guided, non-pharmacologic, and non-invasive medical device featuring an eight-week virtual reality program available through the VA. It integrates cognitive behavioral therapy, mindfulness, and pain neuroscience education. Its immersive modules teach practical self-management strategies to reduce pain and address psychosocial factors that influence mental health. The program is delivered through a series of structured VR sessions designed to promote behavioral skill-building, relaxation techniques, and cognitive reframing of pain experiences (see **Table 1**). The therapeutic environments support breathing practice, attention refocusing, and interactive skill-building exercises, helping Veterans manage pain more effectively while reducing stress and anxiety. The system also provides immediate feedback to users on whether the exercises are being performed correctly.

**Table 1:** RelieVRx sessions.

Chapters	Sessions (6 mins. each)	Descriptions
1	1-7	Breath & Pain
2	8-14	The Mind & Pain Relief
3	15-21	Attention & Distraction
4	22-28	Relaxation
5	29-35	Shaping the Nervous System towards relief
6	36-42	Notice the Body
7	43-56	Journey to Wellness

### 2.2 Adoption of RelieVRx into a pain clinic

**Figure 1:**
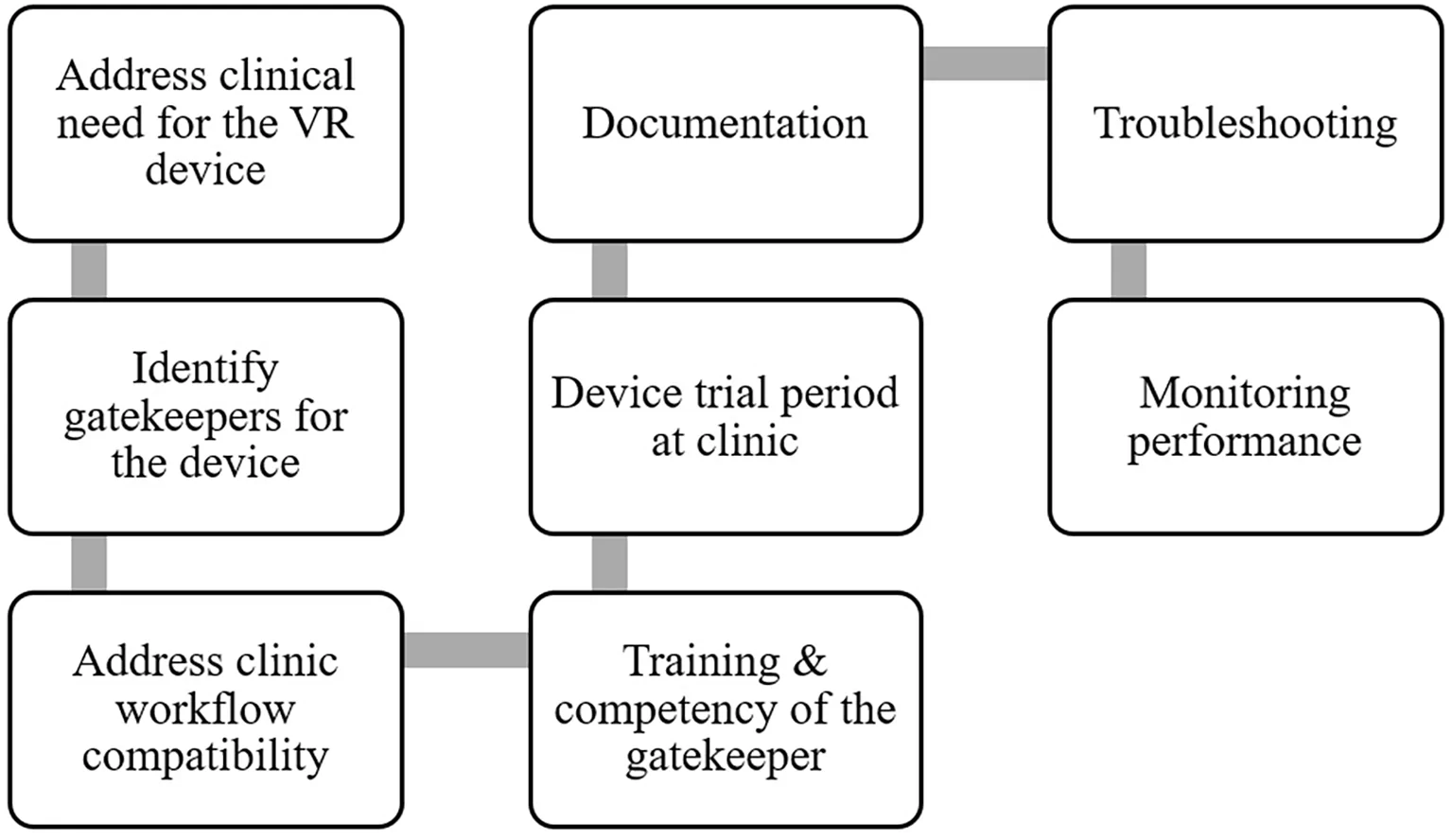
Adoption process of RelieVRx at IPRP.

The adoption process for RelieVRx within the IPRP is illustrated in **Figure 1**. Representatives from AppliedVR, Inc., the developer of RelieVRx, initiated contact with the VA Caribbean Healthcare System (VACHS) and requested a meeting with the IPRP team to discuss the device’s potential application for Veterans receiving care in the program. During this meeting, multidisciplinary clinicians were presented with scientific evidence supporting the device’s effectiveness and provided the opportunity to ask questions regarding its clinical use. Following the presentation, the IPRP team engaged in internal discussions to evaluate the clinical need, appropriateness, and overall goodness of fit of RelieVRx within the existing program structure.

A psychological need was identified related to the impact of pain on patients’ ability to engage in guided imagery–based relaxation skills, which the device could address. Because the device includes cognitive components that complement brief Cognitive Behavioral Therapy for Pain (CBT-P)—one of the psychological interventions offered within the IPRP—Psychology was designated as the discipline responsible for gatekeeping the device. The clinical psychologist subsequently received training on the device and completed a trial period to prepare for orienting patients. A representative from AppliedVR, Inc., also facilitated the acquisition of the device.

Patients were introduced to the device during Session 2 of brief CBT-P, which was intentionally selected because, by that point, patients had already received psychoeducation on pain and therapeutic components relevant to pain management. This allowed the VR content to serve as a reinforcement of previously learned information.

Referral to the device excluded patients with significant cognitive impairment; persistent, elevated PTSD symptoms (especially pronounced startle responses or hypervigilance); psychosis; photophobia; migraines with aura; or balance and mobility impairments that could increase fall risk. Significant cognitive impairment was identified based on documented deficits in orientation, ability to follow multi-step instructions, or other cognitive limitations that could interfere with safe VR participation. Elevated PTSD symptoms were identified using documented clinical history, available standardized assessments, and clinician evaluation of symptom severity. Patients were screened through clinical record review and consultation with treating clinicians. Eligibility decisions were based on clinical judgment regarding the patient’s ability to safely engage in immersive VR exposure. After receiving the device, Veterans had access to customer support from AppliedVR, Inc., for troubleshooting and monitoring of use. The psychologist also assessed device use during subsequent sessions, although not in a standardized manner, an area of opportunity for better tracking of patient progress. Documentation from the psychologist supported both the initial prescription and continuation of the equipment.

### 2.3 Interventions

The RelieVRx program progresses through a structured sequence of phases designed to teach, strengthen, and ultimately generalize pain-management skills. Early sessions focus on foundational concepts, including pain education, basic self-regulation, breathing exercises, guided relaxation, and simple attention-shifting strategies within calming immersive environments. As patients advance, they engage in more active attentional control exercises, expanded breathing practices, and a wider variety of therapeutic environments that encourage generalization of skills across contexts. Mid-program phases emphasize repetition, integration, and smoother transitions between focused tasks and relaxation experiences, helping patients strengthen their ability to regulate attention and physiological responses. Later phases continue refining these skills through interactive focus-shifting tasks, structured breathing practice, and diverse immersive scenarios that promote independence, confidence, and carryover into daily life. In the final phase, patients consolidate all previously learned techniques (e.g., attention control, breathing-based regulation, and relaxation) while relying less on guidance. Psychoeducation is briefly revisited, and practice centers on preparing patients to apply these strategies flexibly in real-world settings. Across all phases, repeated use of VR-supported skills is intended to gradually reshape how individuals respond to pain and enhance their ability to self-manage symptoms beyond the program. All patients underwent RelieVRx 56 sessions. They were offered at the end the opportunity to acquire a sustained device for its use at home even when discharged from IPRP.

## 3 Case description

This case describes a bilingual female Veteran in her fifties, of White Hispanic ethnicity, who served during the Persian Gulf War. She is fully service-connected for several chronic physical and mental health conditions, including respiratory, neurological, and psychiatric diagnoses. The Veteran was initially evaluated by the IPRP for chronic non-malignant pain therapy. She also had a history of fibromyalgia, obsessive-compulsive disorder, and chronic cervical and low back pain. She was married with three adult children and held a master’s degree in finance, though she was retired at the time of evaluation. She received psychiatric services through the VACHS Behavioral Health Integration Program (BHIP), had two psychiatric hospitalizations and one inpatient psychiatric admission, and was prescribed psychotropic medication (duloxetine 20 mg and clonazepam 0.5 mg). She reported drinking alcohol socially and denied tobacco or illicit drug use. She did not present suicidal ideation or perceptual disturbances. She was alert, fully oriented, and independent in activities of daily living. Emotions associated with chronic pain included sadness, irritability, and frustration, along with symptoms of anergia and insomnia. For pain management, she was prescribed baclofen (10 mg) and lidocaine (5%), although she reported that baclofen was not helpful. In addition to receiving RelieVRx, the Veteran participated in brief CBT-P as part of her pain management at the IPRP. Medication was further optimized to include cyclobenzaprine (10 mg), lidocaine (5%), menthol/m-salicylate (10–15%), and pregabalin (50 mg). She successfully completed all 56 sessions of RelieVRx. She reported that the device helped her regulate her mood and decrease her chronic pain and anxiety during and after practice. These improvements were supported by pain and mental health measurement-based care (see **Table 2**). The Veteran noted that the vivid and realistic imagery provided by the device made it easier to engage with and believe the therapeutic content. She described having learned guided imagery in psychotherapy previously, but explained that when in pain, it required significantly more effort to generate mental images on her own effort. She commented on the effort needed to engage with this technique decreasing significantly when using the device. As a result, she requested sustained access to the device.

**Table 2:** Changes in measurement-based care.

Cases	PEG		GAD-7		PHQ-9	
	Baseline	Last session	Baseline	Last Session	Baseline	Last session
Case #1	7	4	–	–	16	5
Case #2	9	3	7	4	9	5
Case #3	5	5	7	5	12	11

This case describes a male Veteran in his late sixties, of White Hispanic background, bilingual and married, who served during the post-Vietnam era. He is fully service-connected for multiple chronic physical and mental health conditions, including PTSD related to military service. He retired from a career in federal service and lived with his family. He had one adult child. The Veteran was enrolled in the IPRP for the second time, and during this admission he was evaluated for severe and debilitating chronic pain that was nonresponsive to therapeutic modalities, including medication. He received psychiatric services through the VACHS Behavioral Health Integration Program (BHIP), had no history of psychiatric hospitalizations, and was prescribed psychotropic medications (aripiprazole 5 mg, duloxetine 30 mg, trazodone 100 mg). He denied alcohol, tobacco, and illicit drug use. He did not present suicidal ideation or perceptual disturbances and was alert and fully oriented. The Veteran reported that TENS treatment provided partial relief, whereas chiropractic care, physical therapy, and acupuncture did not. Emotions associated with chronic pain included frustration, irritability, and anxiety. He required some assistance with basic activities of daily living (ADLs) and full assistance with complex ADLs. For pain management, he was prescribed acetaminophen (325 mg), gabapentin (400 mg), duloxetine (30 mg), lidocaine patches (5%), and ibuprofen (800 mg). In addition to using RelieVRx, the Veteran received Acceptance and Commitment Therapy (ACT) for pain management at the IPRP. Medications were further optimized to include celecoxib (200 mg), pregabalin (50 mg), methocarbamol (500 mg), and diclofenac epolamine patches (1.3%). He completed all 56 sessions of RelieVRx and reported improvements related to feeling calmer and more relaxed. Measurement-based care reflected decreases in pain, depression, and anxiety symptoms (see **Table 2**). The Veteran described being able to achieve a state of relaxation that reduced physical tension and pain intensity while watching the VR content and engaging with guided exercises. He explained that the device helped him focus on the exercises and shift attention away from his pain. Consequently, he requested continued access to the device.

This case describes a bilingual female Veteran in her early sixties, of White Hispanic background, who served during the post-Vietnam era. She is substantially service-connected for multiple musculoskeletal, neurological, and mental health conditions. The Veteran was initially evaluated in the IPRP for osteopenia accompanied by constant foot pain and tingling sensations that worsened with physical activity, along with mild calf cramps during rest. Prior studies suggested possible Tarsal Tunnel Syndrome and refractory pain that limited her ambulation and physical activity. She had previously experienced negative skin reactions to gabapentin (300 mg) and was seeking alternative ways to manage her pain. She lived alone, was single and childless, and worked part-time as a chiropractor. She had no psychiatric hospitalizations and did not use tobacco or illicit drugs. She drank alcohol occasionally in social settings. She was receiving psychotherapy in Primary Care for depression under Cognitive Behavioral Therapy for Depression (CBT-D) and was not prescribed psychotropic medication. She did not require assistance with activities of daily living (ADLs). Emotions associated with pain included frustration and sadness. Other reported symptoms included low energy, insomnia, decreased motivation, poor concentration, anxiety, and irritability. For pain management, she was prescribed lidocaine (5%) and menthol/m-salicylate (10–15%), both of which she found helpful. In addition to RelieVRx, she also received brief CBT-P through the IPRP. Her pain medication regimen was optimized to include capsaicin (0.025%), celecoxib (200 mg), diclofenac (1%), lidocaine (5%), and menthol/m-salicylate (10–15%). She completed all 56 sessions of RelieVRx. She reported a noticeable reduction in perceived pain intensity during and after VR sessions, although this was not reflected in pain measurement-based care (see **Table 2**). She explained that the VR device helped her mentally disengage from the pain, reducing overall bodily tension and promoting a sense of calm and relaxation. Beyond pain, she noticed positive changes in her mood, including reductions in frustration, sadness, and irritability—improvements that were confirmed by mental health measurement-based care (see **Table 2**). As a result, the Veteran requested continued access to the device.

Detailed demographic characteristics of all three participants are provided in **Table 3**. No adverse effects related to VR use, such as cybersickness, were reported by patients during the implementation.

**Table 3:** Patient demographics.

Characteristics	Patient 1	Patient 2	Patient 3
Gender	Female	Male	Female
Age Range	Fifties	Late sixties	Early sixties
Ethnicity	White Hispanic	White Hispanic	White Hispanic
Language	Bilingual	Bilingual	Bilingual
Service Era	Persian Gulf War	Post–Vietnam era	Post–Vietnam era
Marital Status	Married	Married	Single
Children	3 adult children	1 adult child	None
Living Situation	With family	With family	Alone
Employment Status	Retired	Retired	Part-timer
ADL Functioning	Independent	Help with basic and full help with complex	Independent
Medical History	Chronic pain, fibromyalgia, respiratory and neurological conditions	Severe chronic pain	Osteopenia, chronic foot pain
Psychiatric History	OCD	PTSD	Depression
Substance Use	Social alcohol use	None	Occasional alcohol use
Psychiatric Medications	Duloxetine 20 mg, Clonazepam 0.5 mg	Aripiprazole 5 mg, Duloxetine 30 mg, Trazodone 100 mg	None
Pain Medications (Initial)	Baclofen 10 mg, Lidocaine 5%	Acetaminophen 325 mg, Gabapentin 400 mg, Duloxetine 30 mg, Lidocaine 5%, Ibuprofen 800 mg	Lidocaine 5%, Menthol/methyl salicylate 10–15%

## 4 Discussion

These findings should be interpreted as descriptive and reflective of real-world clinical practice rather than evidence of causal effectiveness. All three Veterans completed the full 56-session program and reported improvements in pain-related distress, mood, relaxation, and anxiety, despite differences in pain conditions, functional status, and psychiatric histories. Veterans described VR as easier and more engaging than traditional guided imagery, particularly during periods of heightened pain, as the immersive visuals reduced cognitive effort and facilitated focus on therapeutic exercises. Observed improvements in anxiety and depressive symptoms among two Veterans suggest that VR-based skills training may support affective regulation alongside pain management. These findings suggest that VR-based skills training may influence affective processes such as relaxation, attentional control, and cognitive reframing, which are central to both pain and mental health outcomes. The observed effects are consistent with the cognitive behavioral and mindfulness-based mechanisms embedded within RelieVRx.

In implementing RelieVRx, we found it useful to consider how each procedural step supported its broader clinical integration. Establishing psychology as the clinical gatekeeper ensured that patient selection aligned with behavioral health priorities and enabled appropriate clinical oversight. From there, we examined how the device could be incorporated into routine care, determining that embedding VR within existing CBT workflows allowed clinicians to introduce the intervention without disrupting session structure or treatment continuity. Clinician training and competency verification became central to maintaining fidelity and building confidence in using a novel modality. Before expanding its use, a supervised trial period provided an opportunity to identify challenges, refine procedures, and adjust protocols based on real-world observations. Finally, ongoing monitoring and documentation proved essential for assessing patient engagement and informing decisions about the continued use and clinical utility of RelieVRx.

From an implementation perspective, these cases offer important considerations for pain management clinics exploring the adoption of VR within multidisciplinary services. Factors such as clinical demand, staff training and role delineation, coordination with device vendors, patient inclusion and exclusion criteria, workflow compatibility, trial implementation periods, and standardized documentation procedures are central to successful integration. Across cases, patient-reported benefits were frequently corroborated by changes in measurement-based care, reinforcing the clinical relevance of subjective outcomes.

VR is an increasingly recognized versatile, non-pharmacological tool for pain management in Veterans and military populations by improving pain understanding, reducing pain (23) and anxiety through immersive distraction (15), and enhancing relaxation through guided meditation (24). Advances in VR technology have made it more accessible, portable, and scalable, expanding its potential use from clinical environments to broader and even frontline care settings (25). These attributes may be particularly advantageous in Caribbean healthcare systems, where scalable and transportable interventions may help mitigate disparities in access to specialized behavioral and pain management services. While early evidence shows meaningful benefits for both acute and chronic pain, further research is needed to confirm long-term effectiveness and optimize its integration into routine care.

Notably, VR appeared to enhance guided imagery and relaxation-based skills that may otherwise be disrupted by pain-driven negative imagery (26) or limited imagination capacity (27). Also, within our case report, one patient had history of OCD and people with this diagnosis often struggle with techniques that rely on internal focus or imagination, as these can intensify intrusive thoughts or compulsive mental loops. VR offered an advantage by providing an external, immersive environment that captures attention and reduces the need for internal imagery. Its structured sensory cues help interrupt maladaptive thought patterns and keep the patient grounded in the present (28). In this case, VR acted as a cognitive reset, supporting relaxation without triggering typical OCD-related interference. This illustrates how immersive technologies can be especially useful when traditional relaxation strategies are less effective for individuals with OCD. Beyond immediate reductions in physical tension and emotional distress during VR sessions, the majority of Veterans reported sustained benefits, suggesting that immersive VR may facilitate generalization and continued use of adaptive coping strategies. These observations further indicate that combining immersive VR with brief cognitive behavioral therapy for pain (CBT-P) or acceptance and commitment therapy (ACT) may reinforce skill acquisition and support longer-term therapeutic effects.

Consistent with findings from studies involving broader Veteran populations and general populations, Caribbean Veterans in this case series appeared to experience benefits from VR, particularly in pain and anxiety management (5, 9, 15).While these findings align with existing evidence, they are novel in demonstrating the potential applicability of VR among Caribbean Veterans, a population largely absent from the literature. This case series also advances the limited evidence on the implementation of VR in real-world clinical settings serving bilingual Hispanic populations. Nevertheless, the small sample size (n = 3) necessitates cautious interpretation, and larger studies are needed to confirm these preliminary findings and evaluate the broader impact of VR in this population.

Importantly, this study extends the literature by examining VR implementation among Veterans receiving care within the VA Caribbean Healthcare System, a population rarely represented in digital health and pain management research. Veterans in Caribbean settings may face unique structural and geographic challenges, including limited access to specialty care, fewer non-pharmacologic treatment options, and barriers related to healthcare infrastructure (16, 17, 29). As such, evaluating the feasibility and acceptability of scalable interventions like VR within this context is critical for advancing equitable access to innovative care. Within the Caribbean VA context, these implementation considerations take on added importance, as resource variability and geographic dispersion may influence both adoption and sustainability of new technologies (30). The successful integration observed in this setting suggests that VR-based interventions may be adaptable to diverse healthcare environments and may serve as a practical strategy to extend the reach of interdisciplinary pain management programs.

The present findings align with evidence that VR, particularly RelieVRx, is a feasible and practical addition for chronic pain management in Veterans. Similar to prior work by (31), this case series highlights the successful integration of skills-based VR into clinical care, supporting earlier findings that emphasize VR’s immersive engagement and behavioral skill development (32). While larger trials have distinguished between skills−based and distraction−based VR interventions (33), the current case series was not designed to isolate these mechanisms. Nonetheless, our results expand the literature by highlighting improvements in both pain and psychological well-being, which addresses a specific gap noted in prior studies. Importantly, by including Veterans from a Caribbean VA healthcare system, this study contributes to more inclusive evidence base and underscores the need to evaluate emerging digital therapeutics across diverse and historically underrepresented populations. Overall, our findings support VR as a holistic, non-pharmacologic intervention that targets the physical and psychological aspects of chronic pain in clinical setting. While prior randomized trials have established efficacy under controlled conditions, this case series extends the literature by highlighting real-world implementation in the Caribbean and patient engagement within a multidisciplinary Veteran care setting.

### 4.1 Limitations

This case series includes three patients, and as such, the findings represent only a small, heterogeneous sample. Case series are inherently descriptive and lack the methodological controls necessary to draw causal conclusions. Without randomization, blinding, or a comparison group, it is not possible to determine whether observed improvements were due to the VR intervention itself, the natural course of symptoms, concurrent treatments, or expectancy effects. Therefore, these results cannot be generalized to broader Veteran populations or to individuals with similar pain presentations.

Improvements observed in this case series cannot be attributed solely to RelieVRx, as participants concurrently engaged in multidisciplinary treatments including psychotherapy and medication optimization. Prior sham-controlled trials have demonstrated that non-specific factors, such as expectation and engagement, may also contribute to symptom improvement.

A formal implementation framework such as CFIR was not applied due to the pragmatic, clinician-led nature of this early adoption effort. However, several steps aligned with CFIR domains (e.g., inner setting, intervention characteristics, and process). Future studies should systematically apply implementation frameworks.

Another important limitation is the absence of standardized anxiety assessments for one of the patients and the lack of intermediate assessments throughout the intervention period. Collecting data only at baseline and post-intervention limits the ability to assess the temporal trajectory of symptom improvement. Overall, the findings should be considered exploratory. Larger, pragmatic trials using validated pain and mental-health measures are needed to more rigorously evaluate the effectiveness and long-term impact of VR-based interventions in chronic pain management in real life settings. Given the absence of a control group and the integration of multiple concurrent treatments, causal attribution to the VR intervention is not possible.

## 5 Conclusions

In conclusion, this case series highlights the feasibility of immersive VR as an adjunctive intervention for chronic pain management in Veterans and suggests that immersive technologies may also enhance psychological coping. The observed benefits indicate that VR-based programs such as RelieVRx may simultaneously support pain reduction and emotional regulation by promoting relaxation, attentional redirection, and behavioral skill development. Implementing scalable digital therapeutics such as VR within underserved Caribbean Veteran populations is essential to advancing equitable access to integrated pain and mental health care in settings where geographic and structural barriers may limit availability of specialized services. For clinicians, these findings reinforce the value of incorporating non-pharmacologic, skills-based interventions into comprehensive pain rehabilitation programs, particularly for patients with refractory pain who have not achieved sufficient relief through conventional treatments alone. Collectively, these cases underscore the need for larger, prospective studies to examine underlying mechanisms, clinical effectiveness, and long-term sustainability of VR-supported interventions across diverse Veteran populations. As immersive technologies continue to advance, VR holds promise as a scalable and engaging approach to whole-person chronic pain management in clinical practice.

## Data Availability

The original contributions presented in the study are included in the article/supplementary material. Further inquiries can be directed to the corresponding author.
